# Case report of a seminal vesicle schwannoma involving prostate and bladder: diagnostic pathway and management

**DOI:** 10.1093/jscr/rjaf094

**Published:** 2025-02-25

**Authors:** Paolo Pietro Suraci, Yazan Al Salhi, Giuseppe Candita, Andrea Fuschi, Silvio Scalzo, Onofrio Antonio Rera, Fabio Maria Valenzi, Alice Antonioni, Manfredi Bruno Sequi, Damiano Graziani, Giorgio Martino, Giovanni Di Gregorio, Filippo Gianfrancesco, Luca Erra, Kostika Lako, Alessandro Zucchi, Giorgio Bozzini, Paola Capodiferro, Vincenzo Petrozza, Antonio Carbone, Antonio Luigi Pastore

**Affiliations:** Department of Medico-Surgical Sciences and Biotechnologies Urology, Sapienza University of Rome Faculty of Pharmacy and Medicine, Corso della Repubblica 79, 04100 Latina, Italy; Department of Medico-Surgical Sciences and Biotechnologies Urology, Sapienza University of Rome Faculty of Pharmacy and Medicine, Corso della Repubblica 79, 04100 Latina, Italy; Department of Medico-Surgical Sciences and Biotechnologies Urology, Sapienza University of Rome Faculty of Pharmacy and Medicine, Corso della Repubblica 79, 04100 Latina, Italy; Department of Medico-Surgical Sciences and Biotechnologies Urology, Sapienza University of Rome Faculty of Pharmacy and Medicine, Corso della Repubblica 79, 04100 Latina, Italy; Department of Medico-Surgical Sciences and Biotechnologies Urology, Sapienza University of Rome Faculty of Pharmacy and Medicine, Corso della Repubblica 79, 04100 Latina, Italy; Department of Medico-Surgical Sciences and Biotechnologies Urology, Sapienza University of Rome Faculty of Pharmacy and Medicine, Corso della Repubblica 79, 04100 Latina, Italy; Department of Medico-Surgical Sciences and Biotechnologies Urology, Sapienza University of Rome Faculty of Pharmacy and Medicine, Corso della Repubblica 79, 04100 Latina, Italy; Department of Medico-Surgical Sciences and Biotechnologies Urology, Sapienza University of Rome Faculty of Pharmacy and Medicine, Corso della Repubblica 79, 04100 Latina, Italy; Department of Medico-Surgical Sciences and Biotechnologies Urology, Sapienza University of Rome Faculty of Pharmacy and Medicine, Corso della Repubblica 79, 04100 Latina, Italy; Department of Medico-Surgical Sciences and Biotechnologies Urology, Sapienza University of Rome Faculty of Pharmacy and Medicine, Corso della Repubblica 79, 04100 Latina, Italy; Department of Medico-Surgical Sciences and Biotechnologies Urology, Sapienza University of Rome Faculty of Pharmacy and Medicine, Corso della Repubblica 79, 04100 Latina, Italy; Department of Medico-Surgical Sciences and Biotechnologies Urology, Sapienza University of Rome Faculty of Pharmacy and Medicine, Corso della Repubblica 79, 04100 Latina, Italy; Department of Medico-Surgical Sciences and Biotechnologies Urology, Sapienza University of Rome Faculty of Pharmacy and Medicine, Corso della Repubblica 79, 04100 Latina, Italy; Department of Medico-Surgical Sciences and Biotechnologies Urology, Sapienza University of Rome Faculty of Pharmacy and Medicine, Corso della Repubblica 79, 04100 Latina, Italy; Department of Medico-Surgical Sciences and Biotechnologies Histopathology, Sapienza University of Rome Faculty of Pharmacy and Medicine, Corso della Repubblica 79, 04100 Latina, Italy; Department of Urology, University of Pisa, Via Roma 67, 56126 Pisa, Italy; Division of Urology, Sant'Anna Hospital, San Fermo della Battaglia, Via Napoleona, 60 - 22100 Como, Italy; Sapienza University of Rome Faculty of Pharmacy and Medicine, Department of Medico-Surgical Sciences and Biotechnologies, Radiodiagnostic Unit, Corso della Repubblica 79, 04100 Latina, Italy; Department of Medico-Surgical Sciences and Biotechnologies Histopathology, Sapienza University of Rome Faculty of Pharmacy and Medicine, Corso della Repubblica 79, 04100 Latina, Italy; Department of Medico-Surgical Sciences and Biotechnologies Urology, Sapienza University of Rome Faculty of Pharmacy and Medicine, Corso della Repubblica 79, 04100 Latina, Italy; Department of Medico-Surgical Sciences and Biotechnologies Urology, Sapienza University of Rome Faculty of Pharmacy and Medicine, Corso della Repubblica 79, 04100 Latina, Italy

**Keywords:** schwannoma, seminal vesicle, prostate, bladder, TURB

## Abstract

Seminal vesicle neoplasms are extremely rare. Schwannoma is a benign tumor of the peripheral nerve sheath composed of Schwann cells. Most of these tumors are silent and become symptomatic with compression of adjacent organs and nerves. We present a case of a 65-year-old man with a history of predominant storage lower urinary tract symptoms. Prostate-specific antigen was within normal ranges, and imaging documented a nodular lesion in the prostate, one in the left seminal vesicle and another one adjacent to the right bladder wall. We successfully performed a prostate biopsy and a trans-urethral resection of the bladder to excise and typify the lesion. Histopathology investigation revealed the final diagnosis of schwannoma.

## Introduction

Neoplasms of the seminal vesicles are a relatively rare entity. Among them, benign neoplasms are the most frequent and include fibromas, cystadenomas, leiomyomas, schwannomas, and papillary adenomas. On the other hand, primary malignant neoplasms include adenocarcinoma of the seminal vesicle, sarcomas (leiomyosarcoma, rhabdomyosarcoma, angiosarcoma, Mullerian adenosarcoma-like tumor, cystosarcoma phyllodes), seminoma, squamous cell carcinoma, and extra-gastrointestinal stromal tumors [1]. These are extremely rare, and only sporadic cases are described in the literature. Schwannomas are the most common benign tumors of the peripheral nerves. The aim of this article is to describe the rare case of a patient with a seminal vesicle, prostate, and extra-bladder schwannoma complaining of severe lower urinary tract symptoms (LUTS).

## Case presentation

**Figure 1:**
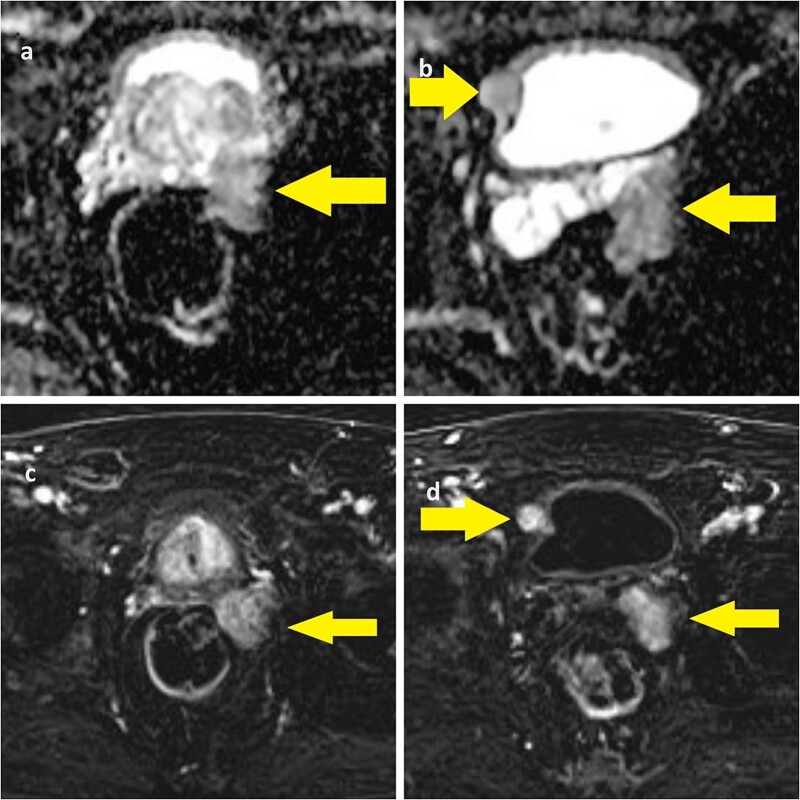
(a) ADC-weighted MRI showing left prostate lobe and left seminal vesicle lesion; (b) T1-weighted MRI showing left seminal vesicle and right paravesical lesion; (c, d) T1-weighted MRI with same lesions.

This is a case report of a 65-year-old man with congenital neurofibromatosis type 2 (NF-2) and acoustic neurinoma. The patient came to our outpatient office complaining of predominant voiding LUTS, namely straining and incomplete bladder emptying. Four months before, patients reported a first aid access due to acute urinary retention, requiring bladder indwelling catheterization, anti-inflammatory drugs, and alfa-blockers therapy (silodosin). The International Prostate Symptom Score was 11, and the patient was mostly dissatisfied with his quality of life related to the urinary condition. A digital rectal examination revealed a prostate with a fibro-elastic consistency, well-defined limits, and no apparent palpable mass. The prostate-specific antigen was within the normal range (2.07 ng/ml), with normal renal function and urinalysis. Uroflowmetry demonstrated an arc-shaped curve with a Qmax of 8.5 ml/s, and the postvoid residual was significant (156 ml). The vesical ultrasound imaging showed a 19-mm wide nodular image adjacent to the right bladder wall. The prostate volume was 74 cc with a third lobe. The patient subsequently underwent pelvic MRI, which showed a bulging mass measuring 34 × 29 × 40 mm located in the left seminal vesicle and the presence of a nodular mass measuring 12 mm in the left prostate lobe with a Prostate Imaging Reporting And Data System (PIRADS) score 4 and a well-delineated nodular mass measuring 19 mm near the right bladder wall (Fig. 1).

**Figure 2:**
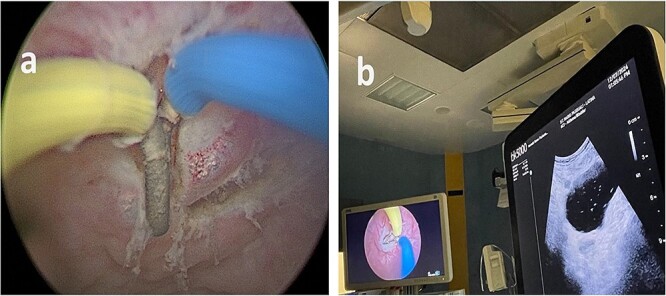
(a) Endoscopic vision during resection. (b) Endoscopic and ultrasound contemporary vision of the lesion.

**Figure 3:**
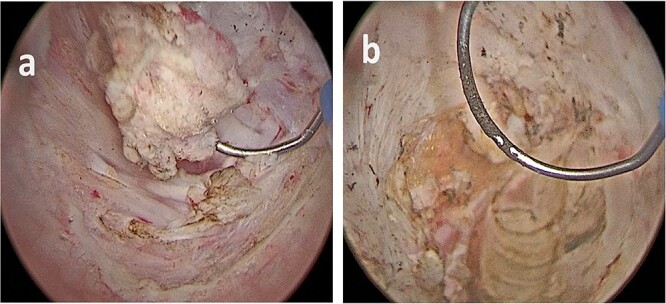
(a, b) Bladder lesion appearing as a nodular intravesical mass, in contrast with typical urothelial bladder cancer, which are commonly endoluminal.

A successful cognitive biopsy of the prostate and left seminal vesicle was performed, immediately after the trans urethral resection of the right bladder lesion. Under general anesthesia, the patient was placed in a dorsal lithotomy position. We started by identifying the cognitive target on the left prostate lobe and proceeded with the biopsy (a random biopsy was also performed). At the urethrocystoscopy, there were no visible lesions in the bladder. With the suprapubic ultrasound, we identified the lesion and we marked it with the Collings loop, and we completed the excision with the standard bipolar loop (Figs 2 and 3).

The lesion appeared different from a typical urothelial bladder cancer (Fig. 3). The total operative time was 55 minutes, no postoperative complication occurred, there was no postoperative pain, and the patient was discharged 2 days later.

**Figure 4:**
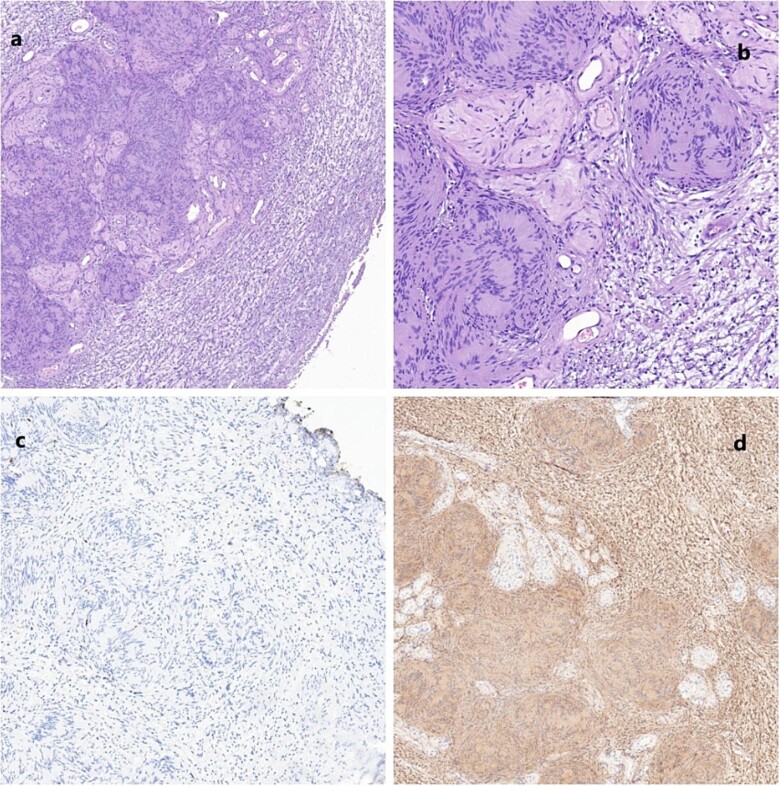
(a) Schwannoma (20×); alternating compact Antoni A areas (center) with nuclear palisades, known as Verocay bodies and loose Antoni B (periphery) areas. Note some characteristic hyalinized blood vessels. (b) Schwannoma (40×). No mitosis, nuclear atypia or necrosis are seen, in accordance with the benign nature of the neoplasm. (c) Very low proliferation index (Ki67); scattered positive cells. (d) Diffuse positive immunoreactivity for S100.

Histopathological and immunohistochemical analysis showed a fusocellular neoplasm composed of cells with elongated nuclei expressing S100 protein. In contrast, CD34 and NF markers were negative and compatible with schwannoma. This appeared on the left seminal vesicle, the prostatic cognitive target, and the bladder lesion (Fig. 4). After surgery, the patient experienced stability in his storage urinary symptoms and showed no signs of recurrence after 1 year of follow-up.

## Discussion

Schwannomas are the most common benign tumors of peripheral nerves [2]. They are encapsulated tumors originating in Schwann cells. Histologically, they are composed of alternating areas of dense cellularity termed Antoni-A regions and areas of myxoid matrix termed Antoni-B regions. Immunohistochemical staining is typically positive for S-100 and negative for CD34, epithelial markers, and smooth muscle-specific actin. [3]. NF is usually positive in other diseases like neuroblastoma or medulloblastoma. Malignant progression is rare; however, a pathological variant (melanotic schwannoma), in which malignant transformation can occur, has been described [3, 4]. Most schwannomas are single lesions and sporadic, affecting individuals of all ages but with a peak occurring between 20 and 50 years, with similar prevalence in both sexes [5]. When these lesions are multiple, they are usually associated with familial syndromes such as neurofibromatosis type 2, schwannomatosis, and Carney complex [6]. They can be found more commonly not only on the head and neck but also on any other body regions, such as limbs, chest, abdomen, retroperitoneum, and pelvis. Schwannoma of the seminal vesicles is extremely rare, with only 12 cases described to the best of our knowledge. The first case was described by Iqbal *et al*. [7]. They are usually asymptomatic and diagnosed incidentally or when the mass becomes large enough to compress adjacent structures. The diagnostic algorithm usually includes an imaging exam (CT scan and/or MRI) to characterize the location, size, and extension of the tumor. Most cases reported to date included a transrectal biopsy for histological characterization. Autieri *et al*. [8] described a case of a transrectal biopsy of a schwannoma of the seminal vesicle complicated with an abscess that needed urgent drainage. Since we considered the possible presence of prostate cancer, the biopsy was essential to obtain a definitive diagnosis. Surgery can be resolutory, but we decided, in accordance with the patient, to adopt clinical surveillance and perform a pelvic RM yearly until new symptoms appear.

## Conclusions

Seminal vesicle tumors are rare, even though prostate and bladder cancer are frequent. Imaging studies play an essential role in the diagnosis in accordance with the patient’s history. However, the histological diagnosis is mandatory to program a future strategy. In this case, conservative approach results in an excellent option for the management of this patient.
